# A Computational Model Based on Multi-Regional Calcium Imaging Represents the Spatio-Temporal Dynamics in a *Caenorhabditis elegans* Sensory Neuron

**DOI:** 10.1371/journal.pone.0168415

**Published:** 2017-01-10

**Authors:** Masahiro Kuramochi, Motomichi Doi

**Affiliations:** 1 Biomedical Research Institute, AIST, Ibaraki, Japan; 2 DAI-LAB, Biomedical Research Institute, AIST, Ibaraki, Japan; 3 Life Science and Bioengineering, Graduated School of Life and Environment Sciences, University of Tsukuba, Ibaraki, Japan; McGill University, CANADA

## Abstract

Due to the huge number of neuronal cells in the brain and their complex circuit formation, computer simulation of neuronal activity is indispensable to understanding whole brain dynamics. Recently, various computational models have been developed based on whole-brain calcium imaging data. However, these analyses monitor only the activity of neuronal cell bodies and treat the cells as point unit. This point-neuron model is inexpensive in computational costs, but the model is unrealistically simplistic at representing intact neural activities in the brain. Here, we describe a novel three-unit Ordinary Differential Equation (ODE) model based on the neuronal responses derived from a *Caenorhabditis elegans* salt-sensing neuron. We recorded calcium responses in three regions of the ASER neuron using a simple downstep of NaCl concentration. Our simple ODE model generated from a single recording can adequately reproduce and predict the temporal responses of each part of the neuron to various types of NaCl concentration changes. Our strategy which combines a simple recording data and an ODE mathematical model may be extended to realistically understand whole brain dynamics by computational simulation.

## Introduction

Mathematical simulation of neuronal activity in the brain helps to describe neuronal encoding for certain animal behaviors and to estimate the circuit dynamics regulating those behaviors. Generating a general theory of neural encoding is a fundamental approach to model the whole brain circuit *in silico*. Simulation of whole brain dynamics can also systematically increase the understanding of the mechanism of neural coding of the sensorimotor integration system in animals [1]. Using calcium imaging methods, observations of neural activities in living animal brains has been performed. This approach enables us to analyze circuit dynamics for perception, memory formation, and decision making dependent on sensory inputs [2–4].

The nematode *Caenorhabditis elegans* has a simple nervous system consisting of only 302 neurons. The patterns of synaptic connectivity in these neuronal cells have been identified by anatomical studies [5, 6]. Real-time calcium imaging from a single-cell level to the whole-brain level can be applied in living worms [7–9], and this can illuminate dynamic responses of individual neurons [10]. This simple, well-characterized nervous system, which is also suitable for imaging analyses, should be quite useful for analyses of neural coding by integrating actual neuronal activity from *in vivo* imaging and computational modeling based on that data. Kato *et al.* successfully applied the integration method and demonstrated a close relationship between sensory-neuronal dynamics and sensory-driven behaviors [11]. However, because this research only treats the activity of cells as a point-unit, it is not clear whether the morphology of the neuronal cells affects to these integration analyses of whole brain activity or not. To handle intrinsic neuronal activities in the brain for computational simulation, a simple model with a smaller number of parameters that can reproduce the various characteristics of neuronal activity is preferable. One of the simple models is Izhikevich’s spiking neuron model which uses only four parameters and can reproduce the spiking and bursting patterns of cortical neurons [12]. Hence, Izhikevich’s model is suitable for exhaustive simulation of neural information processing with a low computational burden [13]. *C. elegans* neurons also show various patterns of responses to several external stimuli [14], however, Izhikevich’s spiking neuron model cannot be applied directly to *C. elegans* neurons since voltage-sensitive sodium channels have not been identified in the *C. elegans* genome [15, 16]. On the other hand, to simplify the analytical method of neural information processing in the worm, a point-neuron model for neuronal activity has been described [11, 17–22]. The point-neuron model neglects the spatial structure of the cell(s) to decrease numerical costs for simulations. To address phenomenological analysis in neural circuit dynamics in the animal, however, each neuronal activity should be analyzed based on its temporal and spatial resolution. Recent studies also indicate that compartmentalized activities at distinct regions of the neurites exist [23–25], suggesting that *C. elegans* neurons should be handled as a multi-unit to build a reliable simulation model. As for a detailed multi-unit model, partial differential equations (PDE) are known to well represent the spatio-temporal activity of single neuron [26]. However, PDE models are numerically or computationally prohibitive for a large neural circuit like a whole brain [27]. Thus, generating a multi-unit model which involves the native morphology and the activity of a *C. elegans* neuron should be quite helpful for simulating whole-brain circuit activity underlying certain behaviors in this animal.

In this study, we first characterized the spatio-temporal calcium activity of a salt-sensing neuron ASER in *C. elegans* with a simple step-down stimulus of NaCl concentration. To build a better mathematical model for intact nervous systems, temporal calcium responses from the dendrite, soma, and axon were observed, and the responses from all parts of the ASER neuron showed similar dynamics. These temporal responses were simple: they peaked within several seconds and decayed slowly until the concentration was increased to a higher one. Based on these actual neuronal-calcium-responses, we constructed a three-unit Ordinary Differential Equation (ODE) model which can predict the spatio-temporal responses of the ASER neuron to various types of NaCl concentration stimuli.

## Materials and Methods

### Strains

The strain used for calcium imaging was *taEx138* [*Pgcy-5:: G-GECO1.2*]. Worms were cultivated on standard NGM agar plates seeded with *E. coli* strain OP50 at room temperature (∼ 22°C).

### Molecular biology and transgenic animals

The *Pgcy-5:: G-GECO1.2* DNA construct for the ASER calcium imaging was generated by first inserting the G-GECO1.2 [28] sequence between the AgeI and *Eco*RI site of the pPD95.79 vector (kind gift from Andy Fire). Then, a 3.0 kb *gcy-5* promoter region was inserted between the SphI and SmaI site of the pPD95.79/G-GECO1.2 plasmid. The resulting plasmid was injected into Bristol (N2) animals at a concentration of 50 ng/*μ*l with the *Plin-44:: mCherry* injection marker using a standard microinjection method [29].

### Calcium imaging

One or two day old adult transgenic worms were used for imaging. Worms were immobilized in a microfluidic device fabricated from polydimethylsiloxane (PDMS) [30]. The microfluidic device was set on an inverted fluorescent microscope (Olympus IX71), and time-lapse images were performed using an ORCA-Flash 4.0 CCD camera (Hamamatsu Photonics) controlled by HCImage software (Hamamatsu Photonics). Recordings were started within 3 minutes after removal from food, and images were captured at the rate of 10 frames/sec. The following compositions of buffers for calcium imaging were used: (in mM) 5 KPO_4_ (pH 6.0), 1 CaCl_2_, 1 MgSO_4_, and 0-50 mM NaCl for the stimulation. All the buffers were adjusted to be 350 mOsmol/L H_2_O with glycerol [31]. The patterns of salt stimulation were automated by using the Perfusion Valve Controller System VC-6M (Warner Instruments) and Arduino microcontroller to control solenoid valves (Arduino SRL) with a pre-generated sequence. A pseudorandom pattern of 50 mM/0 mM NaCl concentration change was generated by Mersenne Twister [32]. We used both ΔF/F_max_ and ΔF/F_0_ for values of fluorescence intensity change. For analysis of temporal dynamics of each region in the cell, the ΔF/F_max_ value was linearly scaled from 0 to 1 with formula (F − F_min_)/(F_max_ − F_min_). F_max_ is the maximum value of the fluorescence intensity, whereas F_min_ is the minimum value of the fluorescence intensity. The ΔF/F_0_ value was used to compare the neuronal activity to the NaCl concentration changes. F_0_ was defined as the average fluorescence in a 5 s window before stimulation. After background subtraction, the total fluorescence intensity was measured from individual regions of interest (ROIs) in the ASER neuron. Photo-bleaching was corrected by fitting a single exponential before and after stimulation and removing the latter by fitted curve.

### A computational model for the spatio-temporal activity in a single neuron

**Fig 1:**
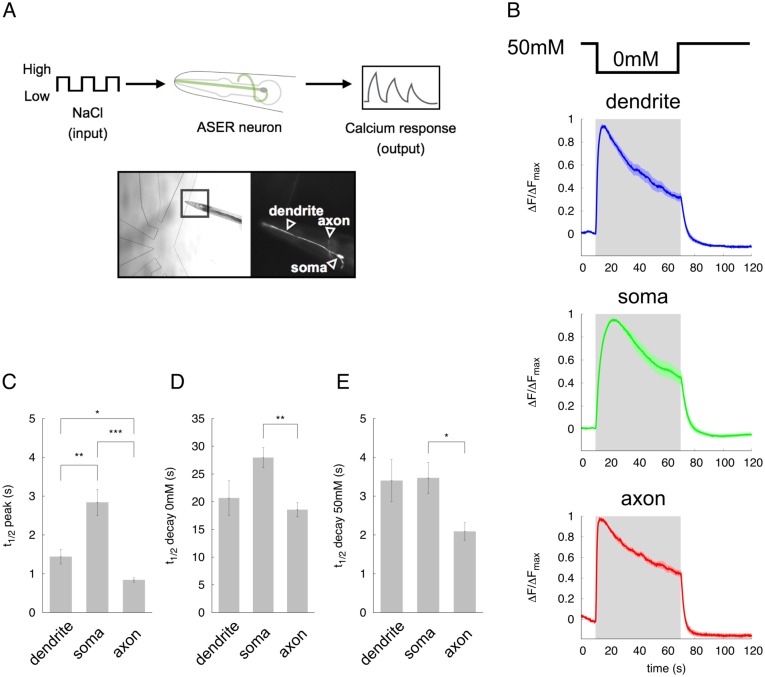
Characteristics of the spatio-temporal response in the ASER chemosensory neuron to step changes in NaCl concentration stimuli. (A) Schematic of the experimental set-up to measure calcium response in the ASER neuron. (B) Normalized calcium dynamics in the dendrite, soma, and axon of the ASER neuron. Gray shading denotes the 60-sec downstep to 0 mM NaCl concentration from 50 mM NaCl (*t* = 10 − 70 s). Solid lines represent average data, and the lightly colored region around each line shows SEM. (C) Time constant to achieve peak response during downstepping of the NaCl concentration. (D) Decay time from peak response to just before the upstep of the NaCl concentration. (E) Decay time back to baseline after the upstep of NaCl concentration. Error bars represent SEM. n = 10, *p < 0.05, **p < 0.01, and ***p < 0.001 by Games Howell test.

Our three-unit ODE model to quantitatively describe spatio-temporal dynamics of a *C. elegans* neuron was formulated as follows:

$$\text{Dendrite}\;\;\tau_{d}\frac{dx_{d}}{dt}=-x_{d}+y_{d}+D\left(W_{d}x_{s}-x_{d}\right)+I\left(t\right),$$ (1)

$$\text{Soma}\;\;\tau_{s}\frac{dx_{s}}{dt}=-x_{s}+Y_{s}y_{s}+D\left(x_{d}+x_{a}-x_{s}\right),$$ (2)

$$\text{Axon}\;\;\tau_{a}\frac{dx_{a}}{dt}=-x_{a}+Y_{a}y_{a}+D\left(W_{a}x_{s}-x_{a}\right),$$ (3)

where *x*_*d*_, *x*_*s*_, and *x*_*a*_ are intracellular calcium dynamics in each region and represent neuronal activities. Time constants are given as *τ*_*d*_ = 1.4 s, *τ*_*s*_ = 3.7 s, *τ*_*a*_ = 1.2 s from the calculation of imaging data (Fig 1). The variable *y*_*i*_ is:

$$\frac{dy_{i}}{dt}=-Ax_{i}\;\left(i=d,s,a\right).$$ (4)

*y*_*i*_ represents a inactivation variable and provides negative feedback to *x*_*i*_. *y*_*i*_ describes the slow delayed-decay during the decrease of NaCl concentration in Fig 1B. A classic leaky integrate-and-fire model, which does not have an inactivation variable *y*, cannot reproduce well this delayed-decay response, because neuronal response converges to a peak response during pulse input [11]. We thus hypothesized that temporal dynamics of this delayed-decay is represented by the inactivation variable which is formulated by the first order differential equation dependent on the self-activity. In addition, we also hypothesized that *y*_*i*_ is zero before and after the stimulus presentation. Without this assumption, the simulated trace shows a hyperpolarized response by negative feedback to *x*_*i*_. However, our imaging results showed that the ASER response was not hyperpolarized after stimulation (Fig 1B). For simplicity, in our model, the *y*_*i*_ works only during the presentation of the input stimulus. The parameter *A* describes the timescale of the inactivation variable *y*_*i*_. Smaller value results in slower inactivation. The parameters *Y*_*s*_ and *Y*_*a*_ describe the magnitude of the inactivation variable *y*_*i*_ to express decay process during the downstep of NaCl stimulation in soma and axon respectively. The magnitudes of decay response during the 0 mM NaCl presentation were different in each unit (Fig 1B). We thus decided to use different parameters for the magnitudes of the inactivation variable in each unit.

The parameter *D* describes a coupling constant in each unit through calcium diffusion. The variables *W*_*d*_ and *W*_*a*_ describe weights in each unit through calcium diffusion from soma. In our model, we hypothesized that calcium can diffuse from soma to both dendrite and axon, and that the diffusion effect from soma depends on the state of dendritic- and axonal-activity. If the dendrite has a high-activity, *W*_*d*_ is smaller than *W*_*a*_. On the other hand, if the axon has a high-activity, *W*_*d*_ is bigger than *W*_*a*_. Details for *W*_*d*_ and *W*_*a*_ setting are shown in Table 1.

**Table 1:** Variables *W*_*d*_ and *W*_*a*_.

	*x*_*a*_ > 0	*x*_*a*_ < 0	*x*_*a*_ = 0
*x*_*d*_ > 0	*W*_*d*_ = |*x*_*a*_|/(|*x*_*d*_| + |*x*_*a*_|) *W*_*a*_ = |*x*_*d*_|/(|*x*_*d*_| + |*x*_*a*_|)	*W*_*d*_ = 0 *W*_*a*_ = 1	*W*_*d*_ = |*x*_*a*_|/(|*x*_*d*_| + |*x*_*a*_|) *W*_*a*_ = |*x*_*d*_|/(|*x*_*d*_| + |*x*_*a*_|)
*x*_*d*_ < 0	*W*_*d*_ = 1 *W*_*a*_ = 0	*W*_*d*_ = |*x*_*d*_|/(|*x*_*d*_| + |*x*_*a*_|) *W*_*a*_ = |*x*_*a*_|/(|*x*_*d*_| + |*x*_*a*_|)	*W*_*d*_ = 1 *W*_*a*_ = 0
*x*_*d*_ = 0	*W*_*d*_ = 0 *W*_*a*_ = 1	*W*_*d*_ = 0 *W*_*a*_ = 1	*W*_*d*_ = 0 *W*_*a*_ = 0

**Fig 2:**
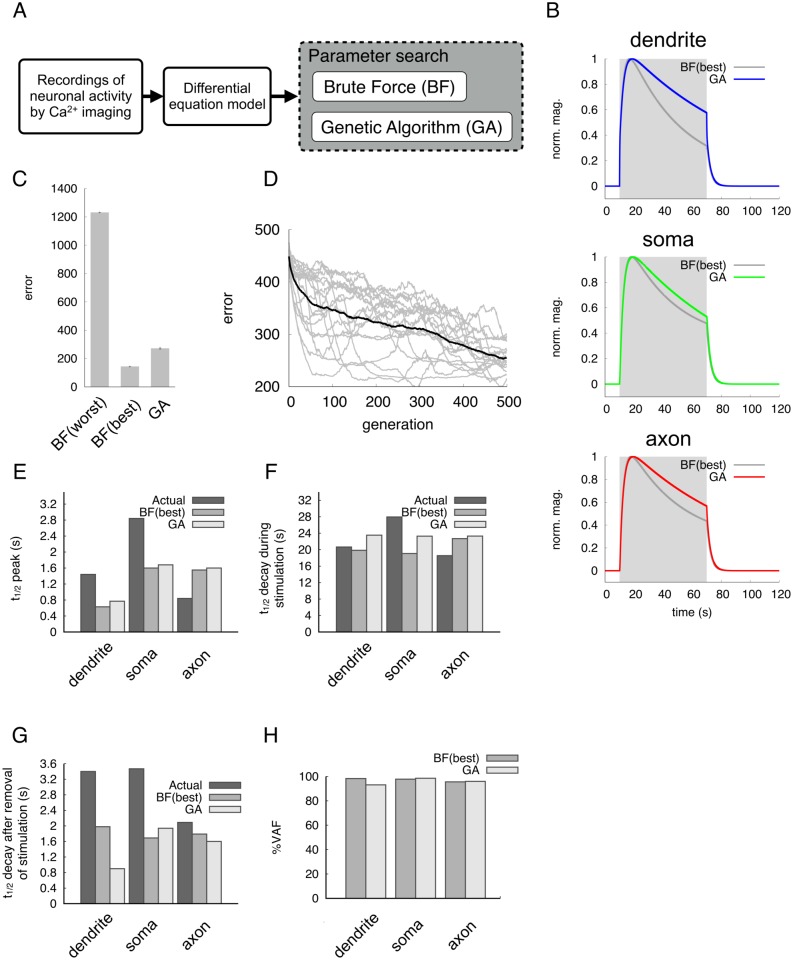
Modeling of spatio-temporal dynamics in the ASER neuron. (A) Experimental flow for modeling of ASER activity. (B) Simulated neuronal activities by two parameter search methods. A 60-second input stimulus was applied to the dendrite. Gray shading denotes the period of input stimulus. Colored lines represent responses estimated by the Genetic Algorithm (GA) method; dark-gray lines represent Brute-Force (BF) method. Magnification is normalized from 0 (baseline) to 1 (peak value). (C) Average fitness from each parameter search, BF or GA. Fitness (error) is a summation of the differences between actual neuronal responses and simulation responses. Small values of fitness indicate that a model reproduces neuronal activity close to the actual response. The best 100 and worst 100 fitness values were calculated in the BF search. In GA search, combinations of free parameters evolved over 500 generations. (D) Evolution process of fitness in GA search. The black line indicates average fitness, and gray lines indicate the individual fitness of eighteen runs. (E) Comparison of the *t*_1/2_ to the peak responses in the dendrite, soma and axon to the input stimulus. ‘Actual’ means the imaging data in Fig 1. (F) Decay times from peak response to the end of input stimulus. (G) Decay times to baseline after removal of input stimulus. (H) Evaluation of simulated responses by VAF index.

**Fig 3:**
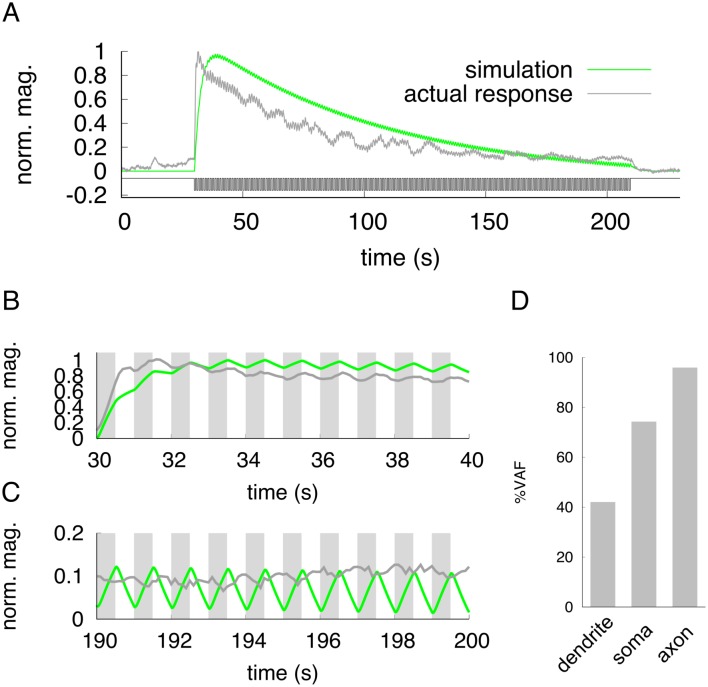
Actual and simulated responses in the ASER neuron to a fast-flickering stimulus. (A) Gray line indicates the actual neuronal response in the soma to a flickering change of 50 mM/0 mM NaCl concentration. The green line shows the simulation of neuronal responses in the soma to a flickering input stimuli. The sequence stimulus input is shown in black. (B) The magnified view around the 30-40 sec interval in (A). Gray shading represents downsteps of NaCl concentration for actual imaging (dark-gray) or simulation (green). (C) The magnified view around the 190-200 sec interval in (A). Gray shading represents downsteps of NaCl concentration for actual imaging (dark-gray) or simulation (green). (D) Evaluation of simulation by VAF index. The response patterns in the dendrite and axon are shown in S2 Fig.

**Fig 4:**
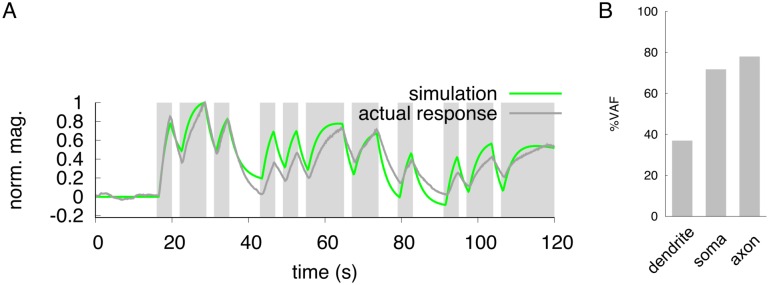
Neuronal responses to a pseudorandom stimulus. (A) Dark-gray line indicates the actual responses in the soma to pseudorandom changes of 50 mM/0 mM NaCl concentration. The green line indicates the result of simulation in the soma to a pseudorandom input stimulus. Gray shading represents the downstep period of NaCl concentration for actual imaging (dark-gray) or simulation (green). (B) Evaluation of simulation by VAF index. The response patterns in the dendrite and axon are shown in S3 Fig.

In our model, *x*_*i*_ and *y*_*i*_ are dimensionless variables, and *D*, *Y*_*s*_, *Y*_*a*_, and *A* are dimensionless free parameters, where *t* is the time. The initial condition of *x*_*i*_ and *y*_*i*_ is zero. The free parameters were optimized from calcium responses to a simple pulse-like stimulation (Fig 1B) by using optimization methods (Table 2). *I*(*t*) is the input stimulus to the downstep in NaCl concentration. For a simple step-like simulation in Fig 2, *I*(*t*) is defined as 1.0 or 0.0, respectively, when the worm is exposed to 0 mM or 50 mM NaCl. For the flickering- or randomized-stimulation in Figs 3 and 4, the ASER neuron was stimulated with *I*(*t*) = 1.5 or 1.0 as the downstep in NaCl concentration, and with *I*(*t*) = 1.0 or 0.5 as an upstep in NaCl concentration to reproduce better performance of simulation.

**Table 2:** Averaged parameters used in our model.

Parameters	BF (best)	BF (worst)	GA
*D*	7.300000 ± 0.180348	0.500000 ± 0.000000	7.1 ± 0.2
*Y*_*s*_	−0.224000 ± 0.00668	−0.14200 ± 0.08181	0.3 ± 0.0
*Y*_*a*_	−0.047000 ± 0.012099	−0.246000 ± 0.086753	−0.2 ± 0.0
*A*	0.105200 ± 0.00244	0.280700 ± 0.000987	0.03 ± 0.0

### Environment of computer simulation

The computational model was implemented using C on a UNIX workstation in which the fourth-order Runge-Kutta method with adaptive time steps was included [33].

### Optimization of free parameters

For modeling, the combination of free parameters *D*, *Y*_*s*_, *Y*_*a*_, and *A* was defined using two methods. We first determined the range of each free parameter that reproduces the corresponding *in vivo* calcium response data (Fig 1B) based on the results of pilot simulations (Table 3). In this pilot simulation, we simulated all the combinations of free parameters (8,000,000) for adequate search ranges of free parameters (see S1 Fig). Next, we applied the Genetic Algorithm (GA) to determine the optimal combination of free parameters in the neuronal models. GA is an evolutionary method used in a heuristic parameter search [34]. An initial population of 100 individuals eight binary character lengths was generated by random selection in each parameter range. Crossover was set to generate the next individuals (offspring) using two randomly-selected current individuals (parents). We defined that a crossover will occur at any randomly-selected points with probability 0.6. The first offspring was generated by combining with the former part of male parent and the latter part of female parent, and vice versa for the second offspring. Bit lengths from male or female parent were dependent on the point of crossover. Randomly-selected bits of individuals were inverted by mutation of the bit strings with mutation probability 0.01. The fitness of all individuals was evaluated by comparing their simulation results (neuronal responses) with the *in vivo* imaging data of the downstep of NaCl concentration for 60 seconds. The fitness function (Euclidean distance between two neuronal activities related to the downstep of NaCl concentration for 60 seconds) was defined as follows:

$$dL=\sum_{t=0}^{120}\left(|f_{d}\left(t\right)-{\hat{x}}_{d}\left(t\right)|+|f_{s}\left(t\right)-{\hat{x}}_{s}\left(t\right)|+|f_{a}\left(t\right)-{\hat{x}}_{a}\left(t\right)|\right),$$ (5)

where *f*_*i*_(*t*) is the calcium responses from actual imaging in each region (Fig 1B). ${\hat{x}}_{i}\left(t\right)$ is the simulated responses in each region. The actual- and simulated-responses are linear scaled from 0 to 1. *dL* is the summation of differences between |*f*_*i*_(*t*)| and $|{\hat{x}}_{i}\left(t\right)|$ for 120 s with interval 0.1 s in all the regions. Smaller values of *dL* indicate better fitness. The populations of parameter sets evolved up to 500 generations. Optimization of parameter set was repeated 18 times using the initial population with different random seeds in GA. Pseudorandom numbers were generated for GA by Mersenne Twister. In addition to GA, we also used the Brute Force (BF) approach to identify a parameter set expected to fit well to the imaging data in Fig 1B. All combinations of free parameters (approximately 1,200,000) were simulated by the BF approach, and their fitnesses were compared to the GA approach. This *dL* was calculated only for the optimization of free parameters from calcium responses to a simple pulse-like stimulation.

**Table 3:** Ranges of free parameters for BF and GA.

Parameters	Range	Step size
*D*	[0, 10]	0.1
*Y*_*s*_	[−1, +1]	0.1
*Y*_*a*_	[−1, +1]	0.1
*A*	[0, 0.3]	0.01

### Evaluation of the model performance

The output responses from the model were evaluated by analyzing the variance accounted for (VAF) values as follows:

$$\text{VAF}=100*\left(1-\frac{\text{var}(x-\bar{x})}{\text{var}(x)}\right).$$ (6)

This VAF index was proposed for the evaluation of model performance as scales relative to the variance of the simulated trace [11, 35]. A model is evaluated as showing good performance when a high VAF is derived from the model. A lower value, however, indicates that the resulting response does not fit the actual imaging data.

## Results

### Characteristics of the ASER calcium response to the NaCl concentration step change

Calcium imaging techniques are suited for evaluating neuronal activities in *C. elegans* as endogenous voltage-sensitive sodium channels have not been identified in the *C. elegans* genome [15, 16]. Instead of the sodium-based classical action potential, *C. elegans* neurons likely to use calcium-based signal amplification for generating currents as in the large nematode *Ascaris* [36–38].

To build a temporal- and spatial-reconstitution model for neuronal activity in *C. elegans*, we analyzed calcium responses in each part of the ASER neuron and tried to understand how salt information is propagated in the dendrite, soma, and axon of this neuron (Fig 1A). The ASER neuron is activated by the decrease in NaCl concentration [39] and mediates chemotaxis behaviors by controlling reorientation movements in response to salt gradients [40–44]. We applied downstep changes in NaCl concentration from 50 mM to 0 mM to transgenic animals expressing G-GECO1.2 protein [28] specifically in the ASER neuron. The duration of 0 mM portion was ranged from 3 s to 60 s, and responses in each part of the neuron were compared (Fig 1B and data not shown). Calcium responses in all the regions of the ASER neuron showed similar patterns of activity; they rose slowly after the downstep of NaCl concentration, and ΔF/F_max_ value reached its peak position several seconds after changing to 0 mM NaCl (Fig 1B and data not shown). After reaching peak response, the calcium response gradually decayed during the downstep to 0 mM NaCl concentration. This decay process during the downstep to 0 mM NaCl concentration lasted until the NaCl concentration was restored to 50 mM (Fig 1B). After the NaCl concentration reached 50 mM (upstep), the ASER activity rapidly returned to the basal, steady state activity. These features of calcium responses which take a few seconds to a peak response and decay slowly during the 0 mM NaCl presentation, were consistent with previous studies [31, 39, 43]. Using the responses to the 60-second downstep stimulation, we quantitatively analyzed their temporal activity (Fig 1C–1E). Response to the peak was fastest in the axon compared to other regions (Fig 1C). The decay responses during the downstep to 0 mM and at the upstep to 50 mM in the axon were also faster than that of soma or dendrite (Fig 1D and 1E). These results indicate that all regions of the ASER neuron are rapidly activated upon the decrease in NaCl concentration, suggesting that signals detected by the cilia rapidly propagate into the dendrite and spread to both soma and axon.

### Modeling of spatio-temporal dynamics in the ASER neuron

Based on the calcium imaging as indices of neuronal activity, we constructed a mathematical model that describes the temporal dynamics of the dendrite, soma, and axon in the ASER neuron (Fig 2A). In our simulation process, the free parameters (*D*, *Y*_*s*_, *Y*_*a*_, and *A*) were optimized by two methods, Brute Force (BF) search and genetic algorithm (GA) (Table 2), and the resulting responses were compared with actual imaging data (Fig 2B–2H). Both parameter-search methods gave quite similar response curves in all unit of the neuron (Fig 2B), and showed low error if best 100 parameter combinations were used in BF (Fig 2C). However, the BF parameter-search using worst 100 data showed quite high error (Fig 2C). Considering the number of trial in parameter search (about 50,000 patterns in GA vs. 1.2 million patterns in BF), GA was more effective than BF when the number of generations increased. Although several discrepancies were observed between actual imaging data and simulation data such as the *t*_1/2_ decay to the 50 mM upstep in the dendrite (Fig 2G), most temporal parameters from simulation seem to be quite similar to those of actual imaging data. This is also confirmed by calculating VAF values in each unit: all the percent VAFs were more than 90%. These results suggest that our three-unit ODE model in combination with the GA parameter optimization can effectively reproduce the spatio-temporal activity of the actual ASER neuron in response to the step change in NaCl concentration.

### Neuronal and computational responses to rapidly-flickering stimuli

Our model was developed by using a single stimulation: downstep to 0 mM from 50 mM NaCl concentration change for 60 seconds. We asked whether our model could predict neuronal responses triggered by other types of stimulations such as weak concentration changes, short-period continuous concentration change, and so on. First, we applied a rapidly-flickering concentration change of NaCl which may be a more realistic model stimulation for naturally-living worms. The worms move their head in 1 s period during for their sinusoidal motion [8]. We changed the concentration at 0.5 Hz, the average rate of worm’s head swing during forward movement. At the onset of the flickering stimulus, gross neuronal activity was observed and this gross activity gradually decreased during the input stimulus (Fig 3A). Similar to the simple one-downstep stimulation, it took several seconds to reach the peak response, and the response decayed slowly until the concentration returned to the basal 50 mM level. At high magnification, we found that calcium concentration in ASER rapidly increased and decreased following the NaCl concentration change (Fig 3B and 3C). This indicates that water-soluble chemosensory neurons can respond to such quickly-repeated stimuli. These quick responses were observed in all unit of the neuron (S2 Fig). The same frequency of input-output stimulation was applied to our ODE simulation model, and we found that the response was quite similar to that of calcium imaging data. The VAF value in the dendrite was relatively low, but in the soma and axon matched to the living cell responses. Thus, our model can predict the neuronal response corresponding to a fast-flickering stimulation.

### Prediction of neuronal responses to unfixed, randomized changes of stimulus

Animals may remain in one field with a fixed environment but walk randomly around to find better conditions. This random movement presents a random change of stimulation to sensory neurons. So we simulated the temporal activity of ASER with a pseudorandom pattern of input stimuli applied (Materials and Methods). The response from our ODE simulation showed a quite similar pattern of activity to the imaging response (Fig 4A). Similar to the quick flickering stimulation, the VAF value in the dendrite was relatively low compared to other unit of the neuron, but the soma and axon responded with almost the same time resolution. We also found that during a long stimulation, the amplitude of neuronal response in the simulation was maintained, but that of actual response gradually decreased (Fig 4 and S3 Fig). These data suggest that our ODE model can effectively predict *in silico* the patterns of neuronal activity mimicking natural behaviors despite the fact that the modeling was constructed using the ASER response to a simple downstep concentration change.

### Neuronal responses to various NaCl concentration changes

**Fig 5:**
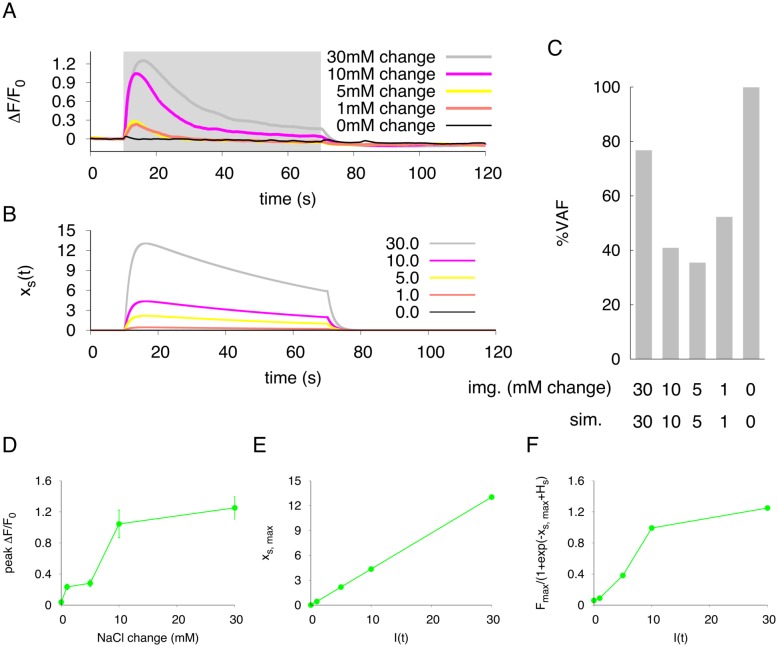
Characteristics of neuronal response to various stimulus changes. (A) Average calcium dynamics in neuronal response (soma) to concentration steps of various sizes from baseline (50 mM NaCl). The ΔF/F_0_ value is indicated to compare the neuronal activity to the NaCl concentration changes. n = 20 (n = 10 in 50 mM change). (B) The simulated responses in the soma activity with our model to input stimuli of various magnitudes. (C) Evaluation of simulation performance are shown in VAF. ‘img. (mM change)’ means the size of NaCl downstep in mM, and ‘sim’ indicates the input stimulus in simulation. (D) The relationship between the size of NaCl downstep and triggered actual peak response in the soma. Error bars represent SEM. n = 20 (n = 10 in 50 mM change). (E) The relationship between the input stimulus (*I*(*t*)) and simulated peak response in the soma. (F) The simulated peak response in the soma are plotted after application of sigmoidal transfer function.

The concentration change of NaCl between 50 mM and 0 mM is much larger than the NaCl gradients encountered by moving worms under living (physiological) environments. We applied various patterns of NaCl concentration change, and validated whether our model could predict neuronal responses to any NaCl concentration changes (Fig 5, S4 and S5 Figs). As shown in Fig 5A, somatic responses to NaCl concentration changes were observed from a 1 mM change to the 30 mM change. The response curves for over 10 mM concentration changes were similar to that of 30 mM change; it takes a few seconds to reach peak response, and the response decays slowly until the next upstep of NaCl concentration is presented to the worms. However, the peak responses were significantly reduced when the NaCl concentration changes smaller than 10 mM change were applied to worms. Although decay process after the peak response in the simulation was slower compared to the actual calcium imaging data, our simulation model also showed the same response curves as the experimental responses. In fact, the VAF values were decreased when the concentration change was less than 10 mM change, suggesting that this model may not be suitable to predict the response to slight concentration changes (Fig 5C). As with somatic responses, we also observed that the dendritic and axonal responses were dependent on the difference in NaCl concentration (S4 and S5 Figs). According to imaging analyses, the simulation model can predict neuronal responses well when the salt concentrations gradients are higher than 10 mM change.

In these analyses, the peak calcium responses were not proportional to the concentration differences in NaCl but were sigmoidal (soma in Fig 5D, dendrite in S4(D) Fig, axon in S5(D) Fig). In simulation, we set the input stimulus to be proportional to the difference of NaCl concentration. The peak responses were proportional to the input stimuli, and those responses did not correspond to the actual responses (Fig 5E, S4(E) and S5(E) Figs). However, the peak transition pattern to the input stimulus seemed to be similar to that of actual neuronal response if a sigmoidal transfer function was applied to the peak neuronal activity *x*(*t*) (Fig 5F, S4(F) and S5(F) Figs). Thus, actual imaging analysis suggests that the relationship between the ASER neuronal activity and NaCl concentration changes is not proportional but sigmoidal.

## Discussion

This study used a calcium imaging method to characterize the spatio-temporal activity in the salt-sensing ASER chemosensory neuron in response to various patterns of NaCl stimulation. By adopting the actual neuronal responses by this calcium imaging, we constructed a novel mathematical model named the three-unit ODE model. Surprisingly, this model successfully reproduced the spatio-temporal activity of this neuron to not only a simple downstep-concentration change but also to the various types of stimuli such as randomized concentration changes (Figs 2, 3 and 4), despite our model was constructed based on calcium responses to a simple sensory stimulation. This finding implies that a computational model in *C. elegans* can be developed relatively easy from the neuronal response to a simple pulse-like stimulation.

The temporal responses of the ASER neuron to the NaCl concentration changes have been well characterized. Our calcium imaging data also showed the simple temporal responses to the 50-0 mM downstep of concentration: a relatively fast activation and slow decay followed by peak response during the downstep of NaCl concentration. Although differences of time constants were observed among three regions, all the responses showed similar temporal dynamics to various types of NaCl stimuli. In addition, compartmentalized activity was not observed in both neurites of this neuron as reported in other *C. elegans* neurons [23–25]. These results suggest that the signal can be rapidly transmitted from the cilia to the axon terminal of this cell. We have also shown that in our imaging analysis, calcium goes up and down faster in the axon (also up faster in the dendrite) than in the soma (Fig 1). What factor does influence the time constant of calcium dynamics in each region? The simple answer may be the volume of regions. In the large space of soma, calcium influx or diffusion into the soma may be slow down, and calcium binding to the G-GECO protein can be slow even if the similar concentration of the reporter molecule exists. Alternatively, the number of voltage-sensitive Ca^2+^ channels on the plasma membrane may not be uniform among these regions. For example, UNC-2, a calcium channel alpha subunit similar to the human P/Q-type calcium channel, has been shown to localize as puncta on the axon of the AWC chemosensory neurons [45]. This kind of heterogenous localization of Ca^2+^ channels has been shown to localize predominantly at neurites, not at soma. On the other hand, upstepping to 50 mM NaCl concentration rapidly inactivated the ASER, meaning calcium exclusion or sequestration from the cytosol by as yet unidentified mechanisms. This fast activation and inactivation mechanism are probably required for proper response of the ASER neuron to a continuous rapidly-flickering concentration change, as we have first shown in this study. Not only ASER, but other chemosensory neurons AWC and ASH are known to respond temporally to a rapidly-flickering stimulus faster than 1.0 Hz, the timescale of one head swing [11]. Those data suggest that most *C. elegans* sensory neurons may potentially respond to fast input stimuli according to worm movement.

Our novel neuronal model is based on data from a simple step-down stimulus applied for 60 seconds, and this model can represent the spatio-temporal activity of the single neuron when the same stimulus was applied (Fig 2). In addition, the simulation also fit well with the actual neuronal response to a short flickering stimulus pattern (Fig 3A and 3B). However, the simulated response did not correspond to real neuronal activity in the peak response magnitude; magnitude of the peak height in the simulation was remained relatively constant during stimulation, but in live animals, it gradually decreased over the one-min stimulation (Fig 3C). This feature was also observed when a pseudorandomized concentration change was used for a stimulation pattern (Fig 4). Similar adaptive responses to fluctuating stimuli in the AWC and ASH neurons are seen in the *C. elegans* chemosensory system [11]. Thus, it is probably true that *C. elegans* sensory neurons have sensory adaptation mechanisms. To improve our ODE model, these adaptive responses should be included by adding several parameters. In addition, our model cannot reproduce quantitative neuronal activity to the temporal responses of each unit for large or tiny changes of NaCl concentrations. The actual imaging data suggests that the response of ASER neuron is not linear to the step changes of NaCl concentration but seems to be sigmoidal (Fig 5D). However, our simulations showed that the relationship between the peak of the neuronal response and *I*(*t*) are a linear to from 0.0 to 30.0 (Fig 5E). In addition, the responses to smaller concentration changes showed weak discrepancies with live imaging responses. Thus, a sigmoidal transfer function to the simulated responses was required to fit simulated peak responses to actual neuronal responses observed in various step changes in NaCl concentration (Fig 5F). This sigmoidal shape of stimulus/response relationship indicates a threshold range in ASER sensitivity, as seen in other *C. elegans* sensory neurons [46, 47]. By examining the threshold range to NaCl concentration change in the ASER neuron or that of other sensory neurons, we will improve our model to quantitatively estimate neuronal responses to various ranges of external stimuli. In addition, this sigmoidal transfer allows to predict the peak responses of ASER neuron caused by various input stimuli. It is also possible to estimate a NaCl-concentration environments from actual neuronal activity, by using our model with sigmoidal transfer.

Several functions in the proposed our neuronal response model should be issued to support the interpretation biophysically. The first issue is the inactivation variable *y*_*i*_ in the eq (4). This variable represents slow delayed-decay during a stimulation (decrease in NaCl concentration). A classic leaky integrate-and-fire model cannot reproduce well this delayed-decay response, because neuronal activity converges to a peak response level during pulse input [11]. Therefore, we supposed that temporal dynamics of this delayed-decay in the ASER neuron might be represented adding an inactivation variable which is formulated by first order differential equation dependent on the self-activity. As expected, the simulated responses were well fit to actual imaging data (Fig 2). A short-period of stimulation such as the 3-second stepdown to 0 mM from 50 mM NaCl concentration does not require this variable, but several *C. elegans* neurons show similar kinds of decay response [11, 24]. Considering the fact that our inactivation variable *y*_*i*_ does not affect to represent the response patterns to short-period stimulations (data not shown), this variable can support to simulate neuronal responses to any types of stimulation. The second issue is on the coupling constant *D*, which is the third term in the eqs (1)–(3). This parameter was set to represent calcium diffusion in each unit of the ASER neuron and works for uniform activities in each unit. Although our model is not represented as a term of calcium influx through voltage-sensitive channels on the soma and axon, calcium responses in each unit are reproduced well. Therefore, the calcium dynamics in the ASER neuron may be largely dependent on calcium diffusion more than calcium influx through voltage-sensitive channel, which may influence on the several parameters of the response curve such as the time constant to the peak response.

Our final goal is to develop a whole brain, neural computation model which is based on *in vivo* imaging of the *C. elegans* nervous system. Although we did not found large differences in neuronal responses of three regions, *C. elegans* neurons should be handled as a multi-unit to establish a reliable simulation for network modeling. The point-unit model is well suited for simulation of circuit analysis, but this model bypasses the morphology of each neuron in the circuit. On the other hand, a detailed multi-unit model, which involves complicated morphology of each neuronal cell, is computationally expensive for large-scale network simulations. In consideration of these restrictions, we constructed a three-unit ODE model for addressing both disadvantages. The single neuron is divided into three units based on its basic morphology, and each unit in our model depends only on time. Our three-unit model is more physiologically reliable than a point-unit model and is easier to handle analytically and numerically than other multi-unit models. Not only three major regions of the ASER neuron, furthermore, we can also apply our three-unit model to reproduce compartmentalized calcium dynamics on neurites, which are reported in several *C. elegans* neurons [23–25]. Although several modifications will be required in the term of diffusion of our equations, we believe extensibility of our modeling to several types of neuronal activity *in vivo*. We also propose GA for the parameter estimation of a large-scale network modeling. In the present study, selected parameters from GA showed compatible responses to those from the best BF samples.

In this study, by integrating *in vivo* imaging and *in silico* simulations, we have successfully constructed a simple phenomenological model for a *C. elegans* neuronal activity. We are also trying to fuse our model with a synaptic integration model to understand the neural information processing for salt-chemotaxis behavior. A graded synaptic transmission has modeled as a sigmoidal function dependent on its presynaptic activity [21, 48, 49]. By simply assuming a small coupling constant *D* in each synaptic region, our model with synaptic integration can estimate the responses in each compartmentalized subcellular region. This may be, unlike other point-neuron models, a strong advantage for whole-brain modeling in *C. elegans*, and also will be helpful to figure out mechanisms of sensorimotor integration in animals.

## Supporting Information

S1 Fig Adequacy evaluation of free parameters. All the combinations of free parameters (8,000,000) were simulated by the eqs (1)–(4) using following ranges: *D*[0, 50]; *A*[0, 10]; *Y*_*s*_[-10, 10]; *Y*_*a*_[-10, 10]. *dL* in the eq (5) was calculated from both simulation results and imaging data (Fig 1). (A) All combinations were classified based on the *dL* value. Smaller *dL* indicates a higher reproducibility. Over half combinations are included into the range over *dL* ≧ 1300. (B) Histgram of *dL* value less than 1,000. The number of combinations is decreased according to smaller *dL* values. The combinations, whose *dL* was less than 800, were analyzed for the adequacy evaluation of free parameters (gray-shading area). (C) Histgram of the *D* values. Selected combinations in (B) were classified based on their *D* values. Gray shading denotes the range of *D* used in our simulation. (D) Histgram of the *A* value. Same combinations in (C) were classified based on their *A* values. Gray shading denotes the range of *A* used in our simulation. (E) Histgram of the *Y*_*s*_ value. Same combinations in (C) were classified based on their *Y*_*s*_ values. Gray shading denotes the range of *Y*_*s*_ used in our simulation. (D) Histgram of the *Y*_*a*_ value. Same combinations in (C) were classified based on their *Y*_*a*_ values. Gray shading denotes the range of *Y*_*a*_ used in our simulation.
(TIFF)

S2 Fig Temporal responses of the dendrite and axon to flickering stimuli with 0.5 s pulse. The actual response (gray) and simulated responses (blue) of the dendrite (A), and the actual response (gray) and simulated response (red) of the axon (B) are shown. Stimulus input sequences are same as in Fig 3.
(TIFF)

S3 Fig Temporal responses of the dendrite and axon to pseudorandom stimuli. The actual response (dark-gray) and simulated responses (blue) of the dendrite (A), and the actual response (dark-gray) and simulated response (red) of the axon (B) are shown. Gray shading represents downsteps of NaCl concentration for actual imaging or simulation.
(TIFF)

S4 Fig Characteristics of dendrite response to various stimulus changes. (A) Average calcium dynamics in neuronal response (dendrite) to concentration steps of various sizes from baseline (50 mM NaCl). The ΔF/F_0_ value is indicated to compare the neuronal activity to the NaCl concentration changes. n = 20 (n = 10 in 50 mM change). (B) The simulated responses in the dendrite activity with our model to input stimuli of various magnitudes. (C) Evaluation of simulation performance are shown in VAF. ‘img. (mM change)’ means the size of NaCl downstep in mM, and ‘sim’ indicates the input stimulus in simulation. (D) The relationship between the size of NaCl downstep and triggered actual peak response in the dendrite. Error bars represent SEM. n = 20 (n = 10 in 50 mM change). (E) The relationship between the input stimulus (*I*(*t*)) and simulated peak response in the dendrite. (F) The simulated peak response in the dendrite are plotted after application of sigmoidal transfer function.
(TIFF)

S5 Fig Characteristics of axon response to various stimulus changes. (A) Average calcium dynamics in neuronal response (axon) to concentration steps of various sizes from baseline (50 mM NaCl). The ΔF/F_0_ value is indicated to compare the neuronal activity to the NaCl concentration changes. n = 20 (n = 10 in 50 mM). (B) The simulated responses in the axon activity with our model to input stimuli of various magnitudes. (C) Evaluation of simulation performance are shown in VAF. ‘img. (mM change)’ means the size of NaCl downstep in mM, and ‘sim’ indicates the input stimulus in simulation. (D) The relationship between the size of NaCl downstep and triggered actual peak response in the axon. Error bars represent SEM. n = 20 (n = 10 in 50 mM change). (E) The relationship between the input stimulus (*I*(*t*)) and simulated peak response in the axon. (F) The simulated peak response in the axon are plotted after application of sigmoidal transfer function.
(TIFF)
