# Supplementary figures and images for: A Computational Model Based on Multi-Regional Calcium Imaging Represents the Spatio-Temporal Dynamics in a Caenorhabditis elegans Sensory Neuron
Source: PLoS One. 2017 Jan 10;12(1):e0168415. doi: 10.1371/journal.pone.0168415 (PMC5224993; doi:10.1371/journal.pone.0168415)

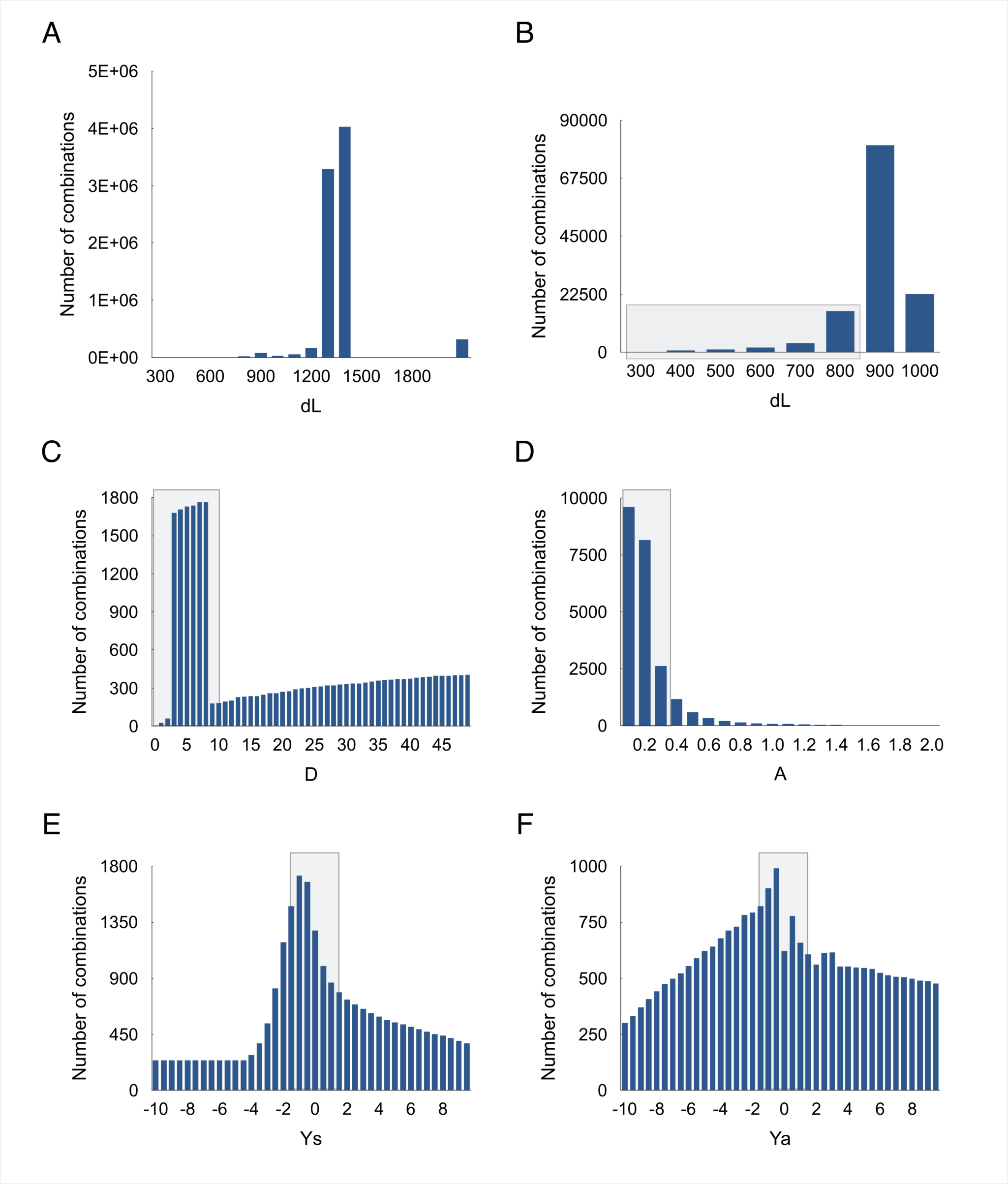

Supplement: S1 Fig — All the combinations of free parameters (8,000,000) were simulated by the eqs (1)–(4) using following ranges: D[0, 50]; A[0, 10]; Ys[-10, 10]; Ya[-10, 10]. dL in the eq (5) was calculated from both simulation results and imaging data (Fig 1). (A) All combinations were classified based on the dL value. Smaller dL indicates a higher reproducibility. Over half combinations are included into the range over dL ≧ 1300. (B) Histgram of dL value less than 1,000. The number of combinations is decreased according to smaller dL values. The combinations, whose dL was less than 800, were analyzed for the adequacy evaluation of free parameters (gray-shading area). (C) Histgram of the D values. Selected combinations in (B) were classified based on their D values. Gray shading denotes the range of D used in our simulation. (D) Histgram of the A value. Same combinations in (C) were classified based on their A values. Gray shading denotes the range of A used in our simulation. (E) Histgram of the Ys value. Same combinations in (C) were classified based on their Ys values. Gray shading denotes the range of Ys used in our simulation. (D) Histgram of the Ya value. Same combinations in (C) were classified based on their Ya values. Gray shading denotes the range of Ya used in our simulation. (TIFF) [file pone.0168415.s001.tiff]

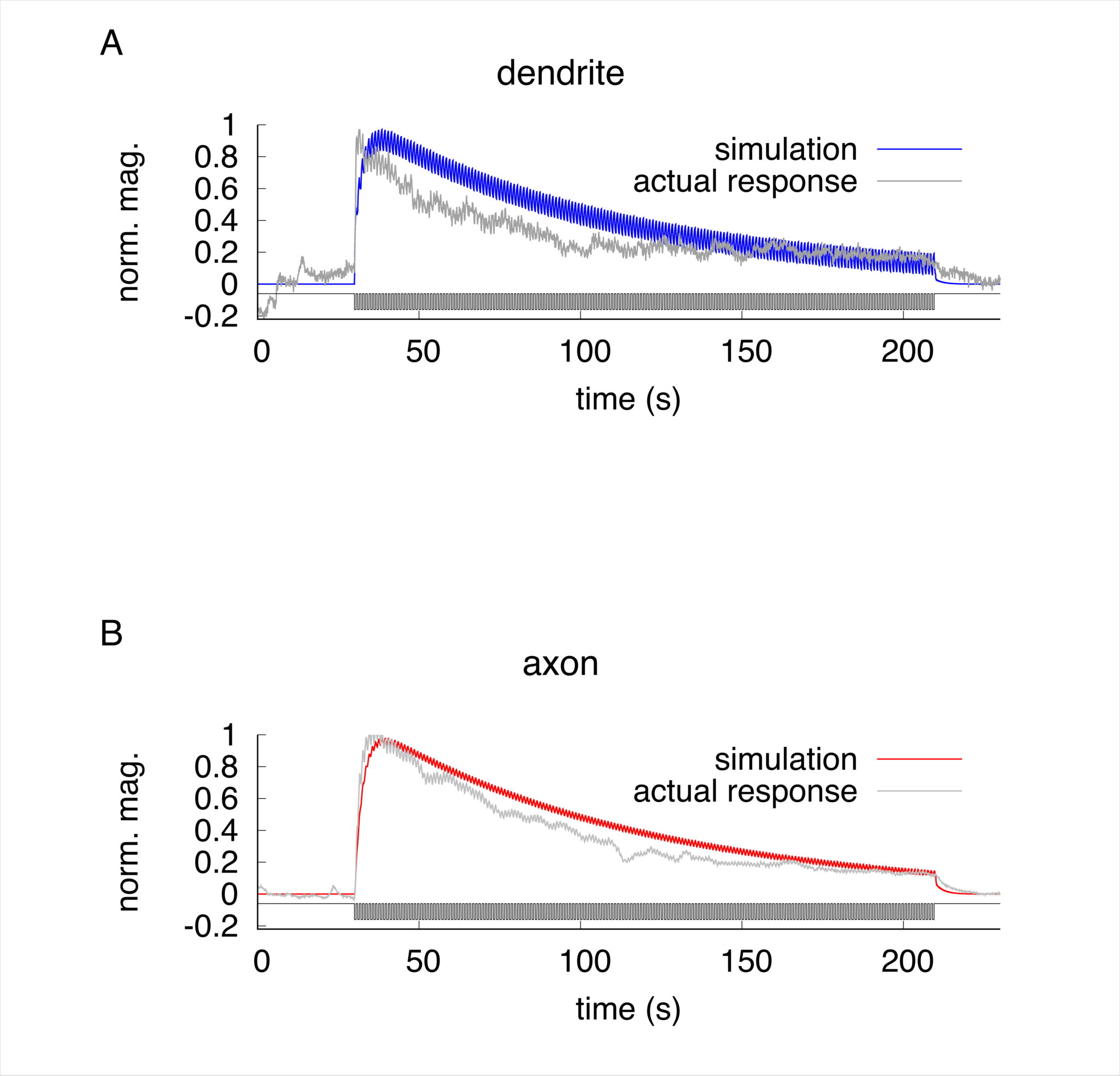

Supplement: S2 Fig — The actual response (gray) and simulated responses (blue) of the dendrite (A), and the actual response (gray) and simulated response (red) of the axon (B) are shown. Stimulus input sequences are same as in Fig 3. (TIFF) [file pone.0168415.s002.tiff]

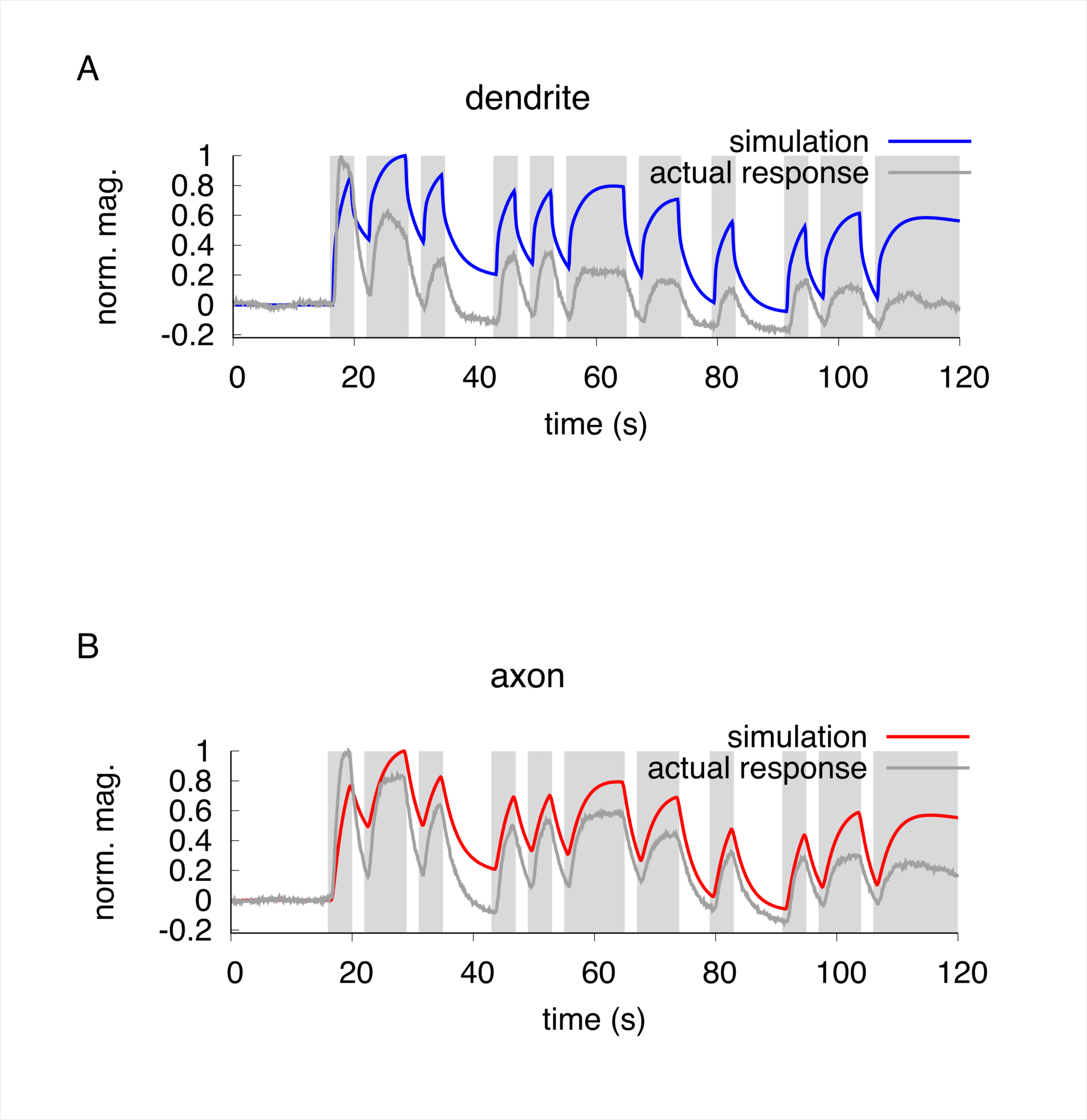

Supplement: S3 Fig — The actual response (dark-gray) and simulated responses (blue) of the dendrite (A), and the actual response (dark-gray) and simulated response (red) of the axon (B) are shown. Gray shading represents downsteps of NaCl concentration for actual imaging or simulation. (TIFF) [file pone.0168415.s003.tiff]

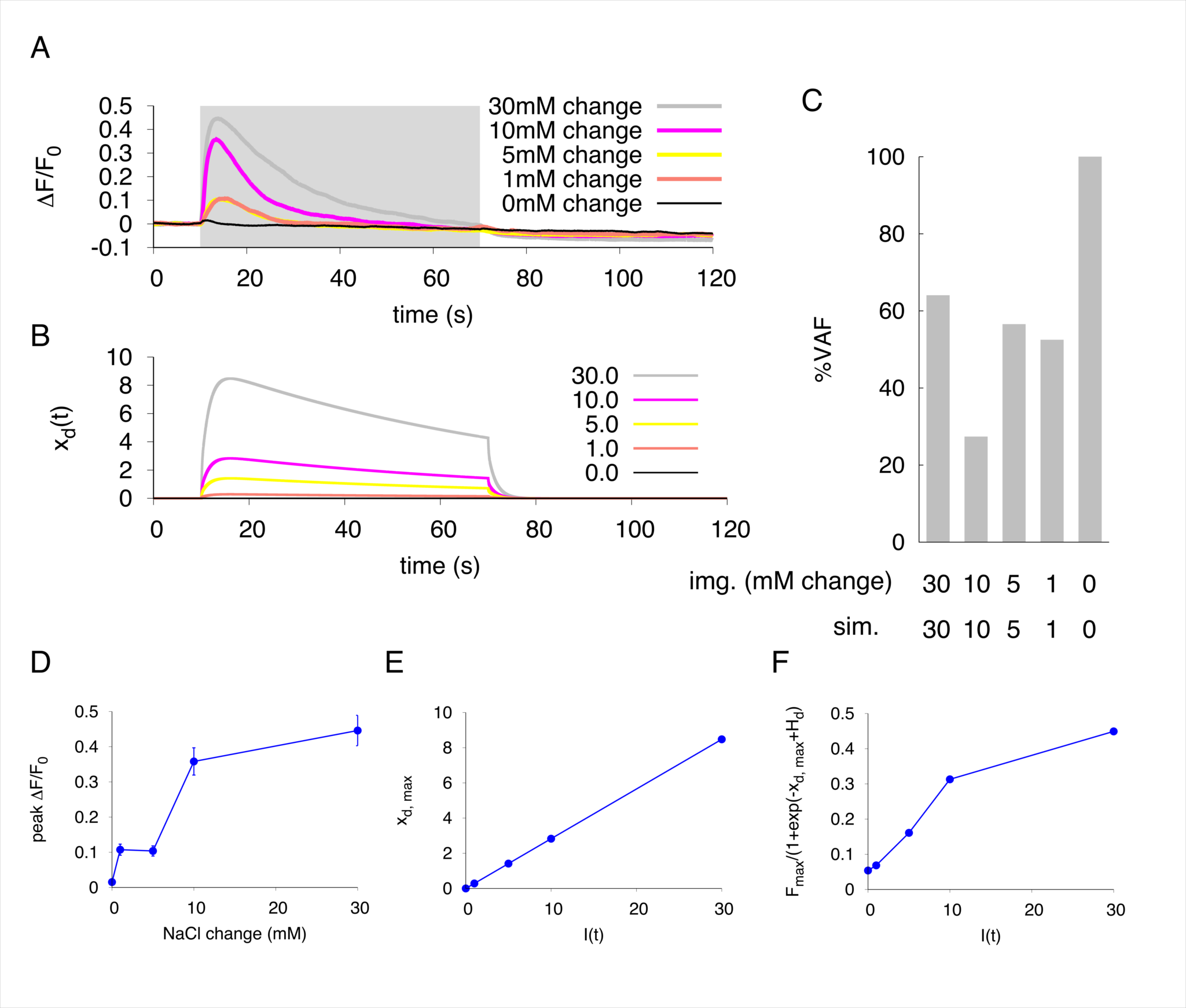

Supplement: S4 Fig — (A) Average calcium dynamics in neuronal response (dendrite) to concentration steps of various sizes from baseline (50 mM NaCl). The ΔF/F0 value is indicated to compare the neuronal activity to the NaCl concentration changes. n = 20 (n = 10 in 50 mM change). (B) The simulated responses in the dendrite activity with our model to input stimuli of various magnitudes. (C) Evaluation of simulation performance are shown in VAF. ‘img. (mM change)’ means the size of NaCl downstep in mM, and ‘sim’ indicates the input stimulus in simulation. (D) The relationship between the size of NaCl downstep and triggered actual peak response in the dendrite. Error bars represent SEM. n = 20 (n = 10 in 50 mM change). (E) The relationship between the input stimulus (I(t)) and simulated peak response in the dendrite. (F) The simulated peak response in the dendrite are plotted after application of sigmoidal transfer function. (TIFF) [file pone.0168415.s004.tiff]

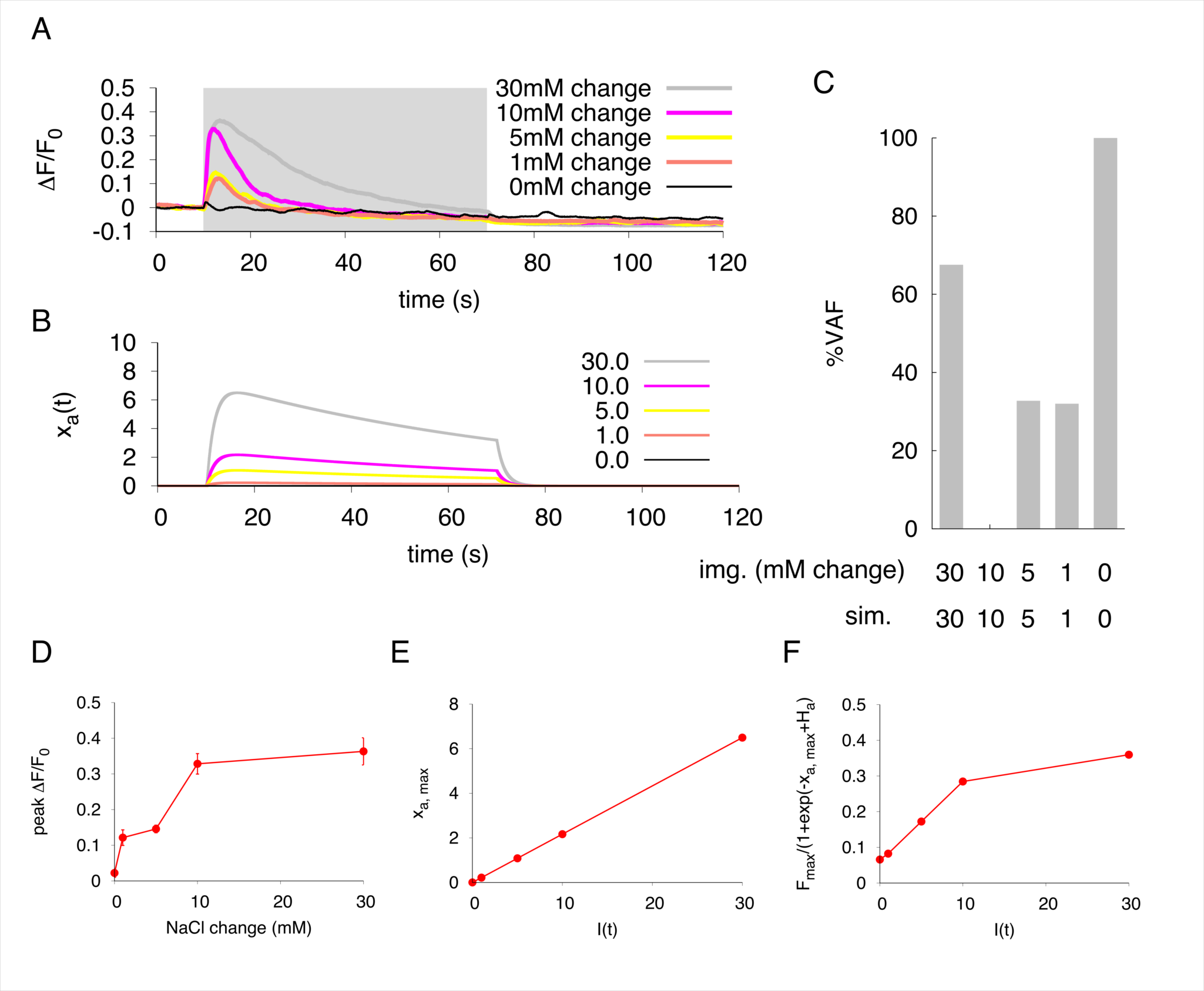

Supplement: S5 Fig — (A) Average calcium dynamics in neuronal response (axon) to concentration steps of various sizes from baseline (50 mM NaCl). The ΔF/F0 value is indicated to compare the neuronal activity to the NaCl concentration changes. n = 20 (n = 10 in 50 mM). (B) The simulated responses in the axon activity with our model to input stimuli of various magnitudes. (C) Evaluation of simulation performance are shown in VAF. ‘img. (mM change)’ means the size of NaCl downstep in mM, and ‘sim’ indicates the input stimulus in simulation. (D) The relationship between the size of NaCl downstep and triggered actual peak response in the axon. Error bars represent SEM. n = 20 (n = 10 in 50 mM change). (E) The relationship between the input stimulus (I(t)) and simulated peak response in the axon. (F) The simulated peak response in the axon are plotted after application of sigmoidal transfer function. (TIFF) [file pone.0168415.s005.tiff]
